# Oxidative Stress Markers and Prediction of Severity With a Machine Learning Approach in Hospitalized Patients With COVID-19 and Severe Lung Disease: Observational, Retrospective, Single-Center Feasibility Study

**DOI:** 10.2196/66509

**Published:** 2025-04-11

**Authors:** Olivier Raspado, Michel Brack, Olivier Brack, Mélanie Vivancos, Aurélie Esparcieux, Emmanuelle Cart-Tanneur, Abdellah Aouifi

**Affiliations:** 1 Infirmerie Protestante, 1 Chemin du Penthod, Caluire-et-Cuire, 69300, France, 33 0624576962; 2 Oxidative Stress College, La Garenne-Colombes, France; 3 Statistique Industrielle Khi² Consulting (KSIC), Bayet, France; 4 Clinical Research and Innovation Department, Infirmerie Protestante, Caluire-et-Cuire, France; 5 Eurofins Biomnis Laboratory, Lyon, France

**Keywords:** oxidative stress, COVID-19, SARS-CoV-2, coronavirus, respiratory, infectious, pulmonary, respiration disorders, hospitalization, machine learning, ML, biomarker, lung, severity, prediction

## Abstract

**Background:**

Serious pulmonary pathologies of infectious, viral, or bacterial origin are accompanied by inflammation and an increase in oxidative stress (OS). In these situations, biological measurements of OS are technically difficult to obtain, and their results are difficult to interpret. OS assays that do not require complex preanalytical methods, as well as machine learning methods for improving interpretation of the results, would be very useful tools for medical and care teams.

**Objective:**

We aimed to identify relevant OS biomarkers associated with the severity of hospitalized patients’ condition and identify possible correlations between OS biomarkers and the clinical status of hospitalized patients with COVID-19 and severe lung disease at the time of hospital admission.

**Methods:**

All adult patients hospitalized with COVID-19 at the Infirmerie Protestante (Lyon, France) from February 9, 2022, to May 18, 2022, were included, regardless of the care service they used, during the respiratory infectious COVID-19 epidemic. We collected serous biomarkers from the patients (zinc [Zn], copper [Cu], Cu/Zn ratio, selenium, uric acid, high-sensitivity C-reactive protein [hs-CRP], oxidized low-density lipoprotein, glutathione peroxidase, glutathione reductase, and thiols), as well as demographic variables and comorbidities. A support vector machine (SVM) model was used to predict the severity of the patients’ condition based on the collected data as a training set.

**Results:**

A total of 28 patients were included: 8 were asymptomatic at admission (grade 0), 14 had mild to moderate symptoms (grade 1) and 6 had severe to critical symptoms (grade 3). As the first outcome, we found that 3 biomarkers of OS were associated with severity (Zn, Cu/Zn ratio, and thiols), especially between grades 0 and 1 and between grades 0 and 2. As a second outcome, we found that the SVM model could predict the level of severity based on a biological analysis of the level of OS, with only 7% misclassification on the training dataset. As an illustrative example, we simulated 3 different biological profiles (named A, B, and C) and submitted them to the SVM model. Profile B had significantly high Zn, low hs-CRP, a low Cu/Zn ratio, and high thiols, corresponding to grade 0. Profile C had low Zn, low selenium, high oxidized low-density lipoprotein, high glutathione peroxidase, a low Cu/Zn ratio, and low glutathione reductase, corresponding to grade 2.

**Conclusions:**

The level of severity of pulmonary damage in patients hospitalized with COVID-19 was predicted using an SVM model; moderate to severe symptoms in patients were associated with low Zn, low plasma thiol, increased hs-CRP, and an increased Cu/Zn ratio among a panel of 10 biomarkers of OS. Since this panel does not require a complex preanalytical method, it can be used and studied in other pathologies associated with OS, such as infectious pathologies or chronic diseases.

## Introduction

Oxidative stress (OS) is an imbalance between the production of free radicals and the body’s ability to neutralize them, leading to damage to cells, proteins, and DNA [1-3]. This is a natural physiological process that is not harmful if it remains balanced, but it can be involved in the onset or worsening of chronic diseases such as cancer [4], metabolic disorders [5], atherosclerosis, [6] and cardiovascular diseases [7,8]. OS can also contribute to nerve cell death and deterioration of brain function, leading to neurodegenerative diseases [9,10].

Healthy lifestyle habits can help reduce OS and its harmful effects, for example, learning to manage emotional stress [11]; performing regular physical activity to promote the production of endogenous antioxidant enzymes [12]; and adopting a balanced diet that is able to provide antioxidants such as vitamins C and E, beta-carotene, and polyphenols and is rich in fruits, vegetables, whole grains, and lean protein sources [13]. Avoiding or reducing exposure to environmental toxins, such as toxic chemicals, cigarette smoke, and air pollutants, can also reduce OS [14].

Furthermore, many respiratory viral infections, including COVID-19, cause death of the infected cells, activation of the innate immune response, and secretion of inflammatory cytokines. All these processes are associated with the development of OS, which makes an important contribution to the pathogenesis of viral infections. COVID-19 is a complex disease in which interaction of the virus with target cells, action of the immune system, and the body’s systemic response to these events are closely intertwined [15].

COVID-19 infection has various levels of pulmonary involvement, with asymptomatic, benign, serious, or even fatal forms, especially when the infected patient has comorbidities such as a history of cancer or malignant hematological diseases; concurrent chemotherapy treatment; severe chronic kidney disease, with or without dialysis; a history of solid organ transplantation or allogeneic hematopoietic stem cell transplantation; chronic polypathologies such as diabetes, high blood pressure, and obesity; or 2 or more organ failures [16]. All of these associated and chronic pathologies will influence the response of the immune system to COVID-19 infection and inflammation, as well as the level of OS present before and during the infection. Recent observational studies have reported that increasing COVID-19 severity may be responsible for a worse prognosis [17] among diabetes patients [18] and increased severity of lung disease [19]. Two meta-analyses showed that supplementation with antioxidants (in the form of vitamins and trace elements) in critically ill patients was associated with decreased mortality and counteracted OS damage [20,21].

The identification of OS biomarkers is essential for early diagnosis of chronic disease, for evaluating treatment efficacy, for monitoring lifestyle interventions, and for understanding the mechanisms involved. The decrease in the blood concentration of thiol proteins is one of the most relevant markers of OS [22]. However, clusters of evidence are emerging from the recent literature showing that levels of biomarkers of oxidative damage in biological fluids can be used for the prediction of measured concentrations of a limited number of exogenous and endogenous antioxidants [23].

Since March 2020, our health care and medical team began to hypothesize that antioxidant supplementation for patients in the intensive care unit (ICU) could improve their prognosis. We were heavily involved in treatment on a daily basis and tried to understand this serious and complex respiratory pathology and treat it as effectively as possible. Our patients benefit from a precise clinical assessment and comprehensive blood biological assays during their initial care and during their hospital stay. Other medical teams have been able to establish scores or panels composed of different blood biological assays to evaluate certain markers of OS and the inflammatory response [16,18], but the measurement and analysis of these biomarkers require complex preanalytical and analytical processes that cannot be carried out during hospitalization in the acute phase, that is, at the patient’s bedside. To identify relevant OS biomarkers, we designed the OXYCOVID study as a feasibility study with the primary objective of testing whether the methodological protocol would be appropriate and feasible in the context of the ICU and ICU admission. The secondary objectives of this study were to identify relevant OS biomarkers associated with the severity of hospitalized patients and to identify whether there is a correlation between the clinical status of hospitalized patients with COVID-19 and severe lung disease at admission using artificial intelligence tools, such as a support vector machines (SVMs). SVMs are used to solve classification problems (eg, category assignment), discrimination problems, and regression problems. They require only a few parameters. The performance of the SVM family of machine learning algorithms is generally as good as, or even better than, neural networks or Gaussian models. These biological assays, if they can be used to determine an OS level, could become a routine element of care, allowing a more precise assessment of the severity of lung lesions in the event of COVID-19 infection.

## Methods

### Study Design

The OXYCOVID study is an observational, retrospective, single-center study based on medical data collected during hospitalization. All adult patients (ie, aged >18 years) hospitalized with COVID-19 at the Infirmerie Protestante (Lyon, France) were included, regardless of the care service they used. Patients were not included if they did not consent to the collection and processing of their personal data, if they were pregnant or breastfeeding, or if they had a protected status (ie, curatorship or other legal protection; deprivation of liberty by judicial or administrative decision). The data were collected from the period from February 9, 2022, to May 18, 2022. The final sample for analysis included 28 patients meeting the inclusion and exclusion criteria. All biological data were recorded in each patient’s digital medical record.

### Data Collection

The data were first collected and then sorted specifically for each patient after checking for the absence of errors and for missing data. After verification, no discrepancies were found and the clinical and biological data were judged to be usable. The data are fully anonymized. The data included sociodemographics (sex and age), comorbidities, and risk factors (weight, height, smoking status, hypertension, diabetes, chronic renal disease, severe chronic respiratory disease, autoimmune disease, immune deficiency, and cancer). COVID-19 vaccines were also recorded, as were data related to the current COVID-19 infection (date of the latest polymerase chain reaction test from a nasopharyngeal swab, variant, and date of first symptoms) and the reasons for hospitalization.

At admission, clinical symptoms were recorded, including the presence of fever (temperature >38°C), cough (yes or no), dyspnea (felt by the patient during the initial questioning), myalgia (felt by the patient during the initial questioning), fatigue (felt by the patient during the initial questioning), diarrhea, oxyge saturation (SpO_2_), oxygenation system used (nasal oxygen therapy, mask oxygen therapy, or Optiflow system), and pulmonary embolism (determined by a thoracic computed tomography angiogram). Biological data were taken from venous blood samples during hospitalization and included complete blood count; levels of urea, creatinine, C-reactive protein (CRP), troponin, D-dimer, ferritin, lactase dehydrogenase, and prothrombin; activated partial thromboplastin time; levels of total bilirubin, gamma-GT, transaminase, albumin, and interleukin-6; and an OxyCheck panel including the following 10 markers: zinc (Zn), copper (Cu), Cu/Zn ratio, selenium, uric acid, high sensitivity CRP (hs-CRP), oxidized low-density lipoprotein, glutathione peroxidase, glutathione reductase, and thiols.

Patients were triaged at admission into 3 mutually exclusive grades. Grade 0 represented asymptomatic patients. Grade 1 represented patients with mild to moderate clinical signs (SpO_2_ >94% in ambient air or with oxygen [maximum 6 L/min], fever, cough, dyspnea, and respiratory rate between 20 and 30 respirations/min). Grade 2 represented the most severe patients, that is, those with severe pneumonia (respiratory rate >30 respirations/min; arterial oxygen saturation <90% despite fraction of inspired oxygen >40% [>6 L/min]) or a rapid increase in oxygen needs (tachycardia [heart rate >120 beats/min]), sweating, agitation, a need for noninvasive ventilatory support with Optiflow (flow rate <60 L/min at 60% fraction of inspired oxygen, ROX index [oxygen saturation/respiration rate index] >3). Grade 2 also included critical patients with severe pneumonia or acute respiratory distress syndrome (index ROX <3 [if the ROX index was <2.5, the patient was intubated]; Optiflow flow rate >60 L/min at 60% fraction of inspired oxygen [if the Optiflow flow rate was >80 L/min at 100% of fraction of inspired oxygen, the patient was intubated]; partial pressure of oxygen in the arterial blood/fraction of inspired oxygen ≤300 mm Hg) and patients with septic shock; acute, life-threatening organ dysfunction related to a known or suspected infection; an altered mental state (delirium or confusion); oliguria; skin mottling; laboratory evidence of coagulopathy, thrombocytopenia, acidosis, or elevated lactic acid; or hyperbilirubinemia.

### Statistical Analysis

Baseline characteristics are reported as counts and percentages for categorical variables and medians and IQR for continuous variables. Each patient was assigned only one grade, so the observations are considered independent. We tested for significant outliers among all the biomarkers, determined if distributions followed a normal distribution using the Shapiro-Wilk test, and used the Levene test for homogeneity of variances. Due to the small sample size, the criteria for outlier values were not strictly applied in the ANOVA. Tukey post-hoc tests were used for multiple pairwise comparisons between severity groups when significant differences were observed in the ANOVA. Finally, an SVM was used to predict assignment for each grade, taking into account the entire analysis profile. Statistical analysis was performed with R (version 4.1.1; R Project for Statistical Computing) and the *tidyverse*, *ggpubr*, and *rstatix* libraries. The level of significance was set at *P*<.05.

Considering that collinearities within our dataset induced by obvious links between biological parameters disrupted traditional methods (eg, logistic regression or the classification method), we used an SVM to predict assignment to each grade for each patient, considering the entire analysis profile. The SVM algorithm is a supervised learning algorithm that uses training data to build a model to predict or classify new observations. SVMs are a machine learning method for nonlinear regression classification [24-26]. Our approach consisted in using the biomarker database as a training dataset for the SVM model (eg, the SVM training dataset). This resulted in a model capable of predicting the probability that a patient would be assigned a specific grade based on the results of a biological analysis, provided that the values did not exceed those used in the initial dataset; that is, the sum of the 3 probabilities was equal to 1. This method resulted in a profile in the form of a graph establishing the link between a combination of biomarkers and the probability of being assigned to the different severity grades. The SVM analysis was performed using JMP Pro (version 17.2; JMP Statistical Discovery).

### Ethical Considerations

Participation in this study was voluntary, and all participants received an individual notice at admission to the ICU, which included informed consent (the individual notice is included in Multimedia Appendix 1). This study was based on data available in the medical record and did not require any additional medical visits or examinations; it also did not lead to any changes in the management or treatment of patients. The data were irreversibly deidentified for the statistical analysis. Participants did not receive any compensation. This study was approved by the ethics committee of Infirmerie Protestante of Lyon (CE-23‐01).

## Results

### Baseline Characteristics of Patients

A total of 28 patients met the inclusion criteria. The median age was 75 (IQR 68.75-80) years, and the patients were mostly male (n=17, 60.71%; Table 1). At admission, 8 patients were asymptomatic (grade 0), 14 had mild- to moderate-severity illness (grade 1) and 6 had severe to critical symptoms (grade 3). The most common reported symptoms were cough and fever.

**Table 1.** Baseline characteristics of the 28 patients hospitalized for COVID-19, including hospitalized patients; socio-demographics, vaccination status, clinical symptoms at admission, and comorbidities.

	All patients (N=28)	Asymptomatic patients (grade 0; n=8)	Mild to moderate patients (grade 1; n=14)	Severe to critical patients (grade 2; n=6)
Sociodemographics				
Sex ratio	1.54	0.6	1.8	5
Male, n (%)	17 (61)	3 (38)	9 (64)	5 (83)
Age (years), median (IQR)	75 (68.75-80)	71.5 (65.5-75.25)	75.5 (68.75-80.25)	71.0 (65.5-78.75)
COVID-19 vaccination, n (%)				
Vaccinated (primary series)	5 (18)	1 (12)	3 (21 )	1 (17)
Unvaccinated	3 (11)	0 (0)	2 (14 )	0 (0)
Unknown	20 (71 )	7 (87)	9 (64 )	5 (83 )
Clinical symptoms at admission, n (%)				
Alteration of the general state	12 (43)	0 (0)	9 (64)	3 (50)
Fever (temperature >38°C)	12 (43)	0 (0)	8 (57)	4 (67)
Cough	10 (36)	0 (0)	9 (63)	1 (17)
Dyspnea (>20 respirations/min)	10 (36)	0 (0)	8 (57)	2 (33)
Myalgia	2 (7)	2 (25)	0 (0)	0 (0)
Diarrhea	3 (11)	0 (0)	3 (21)	0 (0)
Pulmonary embolism	1 (4)	0 (0)	1 (7)	0 (0)
Comorbidities, n (%)				
Diabetes	11 (39)	3 (37)	5 (36)	3 (50)
Hypertension	14 (50)	3 (37)	8 (57)	3 (50)
Cancer	13 (46)	1 (12)	9 (64)	3 (50)

### Descriptive Analysis of the OS Biomarkers

Results for the detection of outliers, the normality test, and the homogeneity of variance are provided in Table 2. The OS biomarkers that met the criteria for the ANOVA were Zn (*P*=.002), Cu/Zn ratio (*P*=.009), hs-CRP (*P*=.02), and thiols (*P*=.004).

**Table 2.** Verification of the conditions for the ANOVA (outlier detection, verification of the normality of values between groups, and verification of homogeneity of variance between groups) and results of a 1-way ANOVA.

	Outlier detection, n			Shapiro-Wilk test ^a^			Levene test^b^	ANOVA
	Grade 0	Grade 1	Grade 2	Grade 0	Grade 1	Grade 2		
Zinc	1	0	0	.97	.97	.66	.35	0.002
Copper	0	0	0	.98	.31	.80	.78	0.343
Copper/zinc ratio	0	1	0	.68	.008	.24	.52	0.009
Selenium	0	0	0	.50	.46	.045	.63	0.303
Uric acid	1	1	0	.02	.19	.60	.89	0.528
High-sensitivity C-reactive protein	0	0	0	<.001	.04	.72	.08	0.017
Oxidized low-density lipoprotein	1	0	0	.18	.70	.33	.55	0.872
Glutathione peroxydase	0	0	1	.18	.51	.03	.99	0.380
Glutathione reductase	0	0	0	.23	.88	.19	.03	0.194
Thiols	1	0	0	.34	.76	.07	.63	0.004

a If the data are normally distributed, the *P* value is greater than .05.

b If the variances are homogeneous, the *P* value is greater than .05.

Statistical differences were observed between the grade 0 and grade 1 patients and between the grade 0 and grade 2 patients for the 3 OS biomarkers, but the difference was nonsignificant between the grade 1 and 2 patients (Table 3).

**Table 3.** Comparison between the severity grades depending on the 3 OS biomarkers using the Tukey post-hoc test.

	Grade 0 versus grade 1	Grade 0 versus grade 2	Grade 1 versus grade 2
Zinc	0.005	0.003	0.609
Copper/zinc ratio	0.012	0.029	0.981
Thiols	0.004	0.019	1

### Predictive Model Using SVM

Table 4 shows the results obtained from the SVM (Multimedia Appendix 2 provides detailed results). The clinical classification and the SVM prediction differed in only 2 of the 28 cases. One of these cases was a patient who was clinically classified as grade 1; he was symptomatic but did not meet the criteria for intubation. The biological analysis profile was abnormal (low thiols [4.1], low zinc, very high Cu/Zn ratio [2.2]). The SVM model classified this patient as grade 2 with a probability of 70.7%. He remained hospitalized for 1 month with active therapy, made good progress, and was discharged. The other patient was initially classified as grade 2; he was strongly symptomatic with high dependence on high-flow oxygen therapy. The biological analysis profile was abnormal (Cu/Zn ratio=3.57). The SVM model classified this case as grade 1 with a probability of 65.8%, probably due to slightly decreased plasma thiol and zinc levels. The clinical course of this patient quickly became favorable.

With a misclassification rate of only 7%, we estimated that the training of the SVM model was good enough and that it was able to predict the grade of patients with COVID-19 infection based on the results of the initial specific biological assays (upon arrival).

**Table 4.** Comparison of the clinically observed grade and the most likely grade (eg, a higher probability of belonging to the grade) predicted by the support vector machine (SVM).

Clinically observed grade	Most likely grade predicted by SVM model (patients), n		
	Grade 0	Grade 1	Grade 2
Grade 0	8	0	0
Grade 1	0	13	1
Grade 2	0	1	5

### Profiles of Biological Results as Illustrative Examples

**Figure 1.**
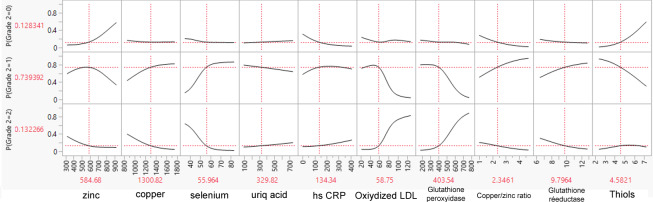
Predicted probabilities of belonging to grade 0, 1, or 2 per biomarker for profile A. Values shown in red indicate the specific values for the profile. P: probability.

**Figure 2.**
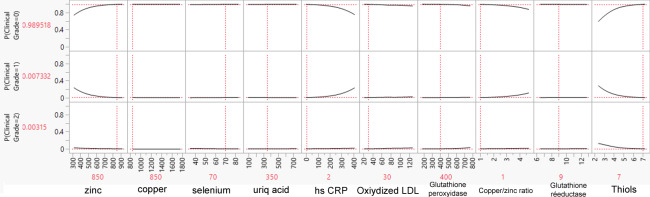
Predicted probabilities of belonging to grade 0, 1, or 2 per biomarker for profile B. Values shown in red indicate the specific values for the profile. P: probability.

**Figure 3.**
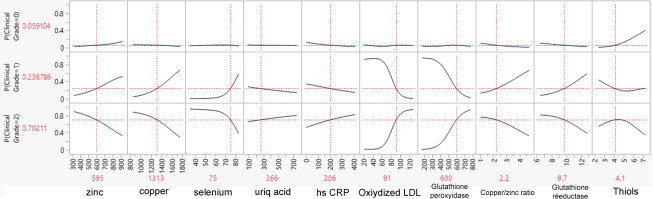
Predicted probabilities of belonging to grade 0, 1, or 2 per biomarker for profile C. Values shown in red indicate the specific values for the profile P: probability.

As an illustrative example, we created 3 biological result profiles (named A, B, and C). These profiles were submitted to the SVM model to determine the probability that they belonged to grade 0, 1, or 2 (Table 5). Profile B had a significantly high Zn level, low hs-CRP, a low Cu/Zn ratio, and high thiols and was predicted to be grade 0. At the opposite end, profile C had low Zn, low selenium, high oxidized LDL, high glutathione peroxidase, a low Cu/Zn ratio, and low glutathione reductase, corresponding to grade 2. Figure 1, Figure 2, and Figure 3 illustrate the predicted probabilities per biomarker and per profile for profiles A to C.

**Table 5.** Examples of 3 different biological profiles and the probability that they belonged to each grade of severity, as determined by the trained support vector machine (Multimedia Appendix 2 provides complete results).

Profile	Zinc (μg/L)	Copper (μg/L)	Selenium (μg/L)	Uric acid (μmol/L)	High-sensitivity C-reactive protein (mg/L)	Oxidized low-density lipoprotein (U/L)	Glutathione peroxidase (U/L)	Copper/zinc ratio	Glutathione reductase (U/g of hemoglobin)	Thiols (µmol/g of protein)	P^a^ grade 0	P grade 1	P grade 2
A	584.68	1300.82	55.96	329.82	134.34	58.75	403.54	2.35	9.80	4.58	12.8%	73.9%	13.3%
B	850.00	850.00	70.00	350.00	2.00	30.00	400.00	1.00	9.00	7.00	99%	0.7%	0.3%
C	595.00	1313.00	75.00	266.00	206.00	91.00	600.00	2.20	9.70	4.10	5.9%	23.9%	70.2%

a P: probabililty.

## Discussion

### Main Findings

Initially, we assumed that antioxidant supplementation for patients in the ICU would reduce the severity of clinical signs and improve their prognosis. The main challenge of this feasibility study was performing a preanalytical and analytical process for measuring OS biomarkers. As a first observational finding, this study identified 3 biomarkers of OS (Zn, Cu/Zn ratio, and thiols) associated with the severity of patients, especially patients with a severity grade of 0 (ie, asymptomatic) or 1 (ie, mild to moderate severity). As a second analytical finding, we found that the SVM model could provide a prediction of the level of severity based on a biological analysis of the level of OS in a relatively limited cohort and during an epidemic of respiratory infectious disease, with only 7% misclassified cases in the training dataset.

The originality of this work is the determination of the grade of clinical severity through the analysis of specific OS biomarkers using a machine learning model. What distinguishes an SVM algorithm from other classical approaches is that when the response is continuous, the fitted models are called a support vector regression (SVR) model. In a typical regression problem, the goal is to fit a model that minimizes the error between a predicted response and the actual response. In an SVR problem, the goal is to fit a model such that the error between a predicted response and the actual response lies in a range of −ε to ε. This allows for more flexible fitting. In JMP Pro, ε is equal to 0.1. The SVR algorithm doubles the data by creating 2 classes, Y + ε and Y − ε. Then, the same algorithm used for the classification problem is also used for the prediction problem (ie, SVR).

Maximization in SVM algorithms is done by solving a quadratic programming problem. In JMP Pro, the algorithm used by the SVM platform is based on the sequential minimal optimization (SMO) algorithm introduced by John Platt in 1998 [27]. Typically, the SVM quadratic programming problem is very large. The SMO algorithm divides the overall quadratic programming problem into a series of smaller problems. The smaller problems are solved analytically rather than numerically, which means that they produce closed-form solutions. Therefore, the SMO algorithm is more efficient than solving the overall quadratic programming problem. While this univariate analysis did not find any differences between grade 1 and grade 2, the trained SVM model estimated the severity grade from the same OS biomarkers; this could have a significant impact in the management of patients at risk of sudden respiratory decompensation. Although the power of the model would benefit from being improved with clinical and biological data from a larger cohort, this approach could be applied to other types of epidemics and other biological assays, as well as to the investigation of other diseases.

### Comparison With Prior Work

We know that moderate and severe forms of COVID-19 are associated with high OS and a low total antioxidant level, which is measured with complex preanalytical and analytical methods [28,29] that are not generally used in all laboratories, have long delays in receiving results, and are not compatible with management in ICUs [30]. Moderate and severe forms of COVID-19 are accompanied by an increase in inflammatory biomarkers, such as CRP, procalcitonin, the neutrophil to lymphocyte ratio, and hemostasis parameters (activated partial thromboplastin time, prothrombin time, D-dimers, and fibrinogens) [29]. The natural correction of low levels of endogenous antioxidants is slow, with a tendency to recover 3 months after hospital discharge [28].

High OS is not only specific to severe disease or infection, but positively influences their severity. A basic infectiology study with a porcine animal model of infection with the avian-type H1N1 European swine influenza virus has shown that prior infection of animals with mycoplasma generates a high level of OS, increasing the severity of influenza and reducing animal performance; host responses could be influenced by diet [31].

The systemic OS status, determined using many biomarkers, was still significantly increased in recovered COVID-19 patients during the recovery phase. Nonhospitalized individuals with COVID-19 presented signs of systemic OS, which is longitudinally associated with the development of post–COVID-19 condition. Regular assessment of plasma thiol levels as a monitoring biomarker and supplementation in cases of deficiency might be potential therapeutic targets at the onset of post–COVID-19 condition [32].

From the beginning of the epidemic, plasma thiol assays at admission were a promising tool to predict ICU admission in patients with COVID-19 [19]. The principles of nutrition for patients in intensive care follow validated recommendations with a high level of evidence [33]. Faced with inflammatory syndrome and significant catabolism, caloric and protein intake must be sufficient to combat muscle wasting and the risk of infection. In products intended for parenteral or enteral nutrition, specific micronutrients or macronutrients intended to modulate inflammatory processes and optimize the body’s immunological or metabolic responses are added, such as the antioxidants β-carotene, vitamin A, vitamin C, or vitamin E, in order to combat OS [34], which plays a central role in the development of major inflammatory states and visceral failure through the production of reactive oxygen species (free radicals), which influence the consumption, distribution, and level of antioxidants [35]. Lowered levels of antioxidants are common and are associated with an increase in morbidity and mortality [36]. Two meta-analyses showed that supplementation with antioxidants (eg, vitamins and trace elements) among ICU patients is associated with a reduction in mortality [20,21] and a reduction in mechanical ventilation time [20], but two more recent meta-analyses showed that the evidence for these benefits remained questionable because they were measured in small study populations and with highly variable cocktails of antioxidant nutrients [23,37]. Currently, it is not recommended to provide massive doses of antioxidants to patients treated in intensive care.

### Strengths and Limitations

The main limitation of this study is the sample size, with less than 10 patients in the grade 0 and grade 2 groups. Despite the small number of patients, we observed a statistical difference between grade 0 and grade 1 patients in terms of biomarkers associated with severity. The small sample size was caused by a short study inclusion period due to the hospital context and the ongoing crisis, which was not suited to the inclusion of more patients or to the inclusion of other hospitals. The medical and nursing team has continued to collect relevant clinical and biological data concerning patients treated for COVID-19 infection since the end of the study. These new data could be aggregated with those collected during this study and analyzed with an SVM in order to improve the performance of the severity stage prediction. As a beneficial side effect, this study allowed us to test the ability of the clinical research team to study OS biomarkers and identify covariates and risk factors for further investigation after using propensity scoring to adjust for comorbidities and sociodemographic variables of patients.

As early as 2020, applying the SVM method to COVID-19 was reported in the scientific literature, but it was mainly used for diagnosis and prediction of severity in a radiological manner and as a scanner [26]. The prediction of severity stage by SVM has been extended to the analysis of biomarkers classically used in inflammatory and infectious pathologies [27,28] but has not provided a consensus or an infallible score to predict the level of severity. To our knowledge, this study is the first to show the SVM method being used for predicting the severity stage of COVID-19 with pulmonary involvement.

An important point to consider before generalizing the use of prediction scores based on this panel of blood biological OS analyses is the dosage method and standardization of reference values for each analysis and between each laboratory.

### Conclusions

In patients with COVID-19 infection, moderate to severe symptoms were correlated with a lowered Zn level, a lowered plasma thiol level, an increased hs-CRP level, and an increased Cu/Zn ratio in a panel of 10 OS biomarkers not requiring complex preanalysis (Zn, Cu, Cu/Zn ratio, selenium, uric acid, hs-CRP, oxidized LDL, glutathione peroxidase, glutathione reductase, and thiols). Applying a machine learning method (an SVM) to the results of this same panel to evaluate the OS level in a retrospective cohort allowed prediction of the level of severity of pulmonary damage in hospitalized patients with COVID-19. Since this panel does not require a complex preanalytical method, it can be used and studied in other pathologies associated with OS, such as infectious pathologies or chronic diseases. Intensive care teams are accustomed to monitoring and managing major inflammatory symptoms, and although it is likely that they need to augment their practices to manage OS and the supply of antioxidants, the biological exploration of OS requires highly complex preanalytical and analytical processes that, to date, are highly limiting factors in the development and dissemination of relevant OS assessments. The fragility and thermolability of certain biomarkers means that any redox reactions that might take place in the sample must be halted; thus, the mandatory use of −80 °C conditions throughout the preanalytical phase makes analysis complex and in some cases even technically unfeasible. We have therefore developed an OS panel with 10 biomarkers that overcomes all these constraints without compromising scientific relevance by performing an assessment of OS based on a series of biomarkers that do not require complex preanalysis (simple freezing at −20 °C); these biomarkers were selected with a machine learning approach in 2009 [23]. The results from this panel showed real significance in estimating OS with robust and reproducible results. Iterative training of this SVM model with a biomarker panel in a larger cohort of patients with OS would give it more power and precision. By determining the ideal weighting of each of the biomarkers with a scientifically validated algorithm, we could obtain a score to estimate the level of OS. A Belgian intensive care team assessed total antioxidant capacity with an electrochemical method (eg, the Total Antioxidant Power technology) rather than by the analysis of biological samples (blood, saliva, or urine), finding that it had potential as a less expensive and much faster alternative to the individual analysis of biomarkers linked to pro-oxidants [30]. This would allow intensive care teams to monitor and respond in real time to systemic OS in their patients.

The World Health Organization has already announced the very probable risk of the appearance of a new epidemic caused by new types of severe acute respiratory syndrome or by the “humanization” of the avian influenza A (H5N1) virus in the years to come [38]. Given the contagiousness and the risk of decompensation, particularly respiratory decompensation, or even death from these viral infections, considering systemic OS in addition to the inflammatory syndrome could be a determining factor in the effectiveness of care in the future. The early and systematic use of machine learning models such as SVMs in clinical, radiological, and biological assessments at the start of an epidemic could provide a better understanding of the functioning and mechanisms of novel pathogens and reduce human loss of life. Sharing of results between different care centers with dedicated databases would accelerate learning and improve the accuracy of these statistical tools.

From a public health perspective, individuals are subject to a range of exposures through the environment (air pollution, solar radiation, and temperature variations) and lifestyle (lack of sleep, psychological stress, tobacco, and poor diet); this is summarized in the term “exposome.” This directly impacts the OS level and therefore the body’s ability to defend itself in the event of infection. Taking action to maintain a healthy environment and lifestyle would help mitigate the severity of diseases such as COVID-19 [39].

## Supplementary material

10.2196/66509 Multimedia Appendix 1 Individual notice information.

10.2196/66509 Multimedia Appendix 2 Support vector machine results from 28 patients, including observed oxidative stress biomarkers and the probability of patients being assigned severity grades 0, 1, or 2.

## Abbreviations

CRP: C-reactive protein
Cu: copper
hs-CRP: high-sensitivity C-reactive protein
ICU: intensive care unit
OS: oxidative stress
ROX index: oxygen saturation/respiration rate index
SMO: sequential minimal optimization
SpO_2_: oxygen saturation
SVM: support vector machine
SVR: support vector regression
Zn: zinc
