# Supplementary material for: Oxidative Stress Markers and Prediction of Severity With a Machine Learning Approach in Hospitalized Patients With COVID-19 and Severe Lung Disease: Observational, Retrospective, Single-Center Feasibility Study
Source: JMIR Form Res. 2025 Apr 11;9:e66509. doi: 10.2196/66509 (PMC12007842; doi:10.2196/66509)
Supplement: Multimedia Appendix 1 [file formative-v9-e66509-s001.docx]

| 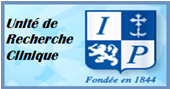   \| **INDIVIDUAL NOTICE INFORMATION**  For patients participating in research involving human subjects \| \| --- \| |
| --- | --- |
|  |
| **OXYCOVID study**  **Single-center retrospective observational study evaluating the correlation between the clinical course of unselected hospitalized patients with moderate, severe or critical COVID-19 pneumonitis AND the course of a panel of specific “Oxidative Stress” biomarkers.** |

**Sponsor :**

INFIRMERIE PROTESTANTE DE LYON

1-3 chemin du Penthod

69300 CALUIRE-ET-CUIRE

Tél : +33 (0)4 26 29 79 27 (Clinical Research Unit)

Email : [recherche-clinique@infirmerie-protestante.com](http://?)

Dear Sir/Madam

Following your hospitalization for a COVID-19 infection, Doctor RASPADO Olivier, the investigating physician for this study, is inviting you to take part in the OXYCOVID study.

To help you make your decision to participate in this study, it is important that you understand why this study is being carried out and what it involves. This document has been designed to provide you with the information you need to understand the benefits of the study and how it will work.

If you have any questions, you can ask them to the investigating doctor who proposed this study. Before making a decision, you may also wish to discuss your participation with your treating physician and those close to you.

It is also important to know that your participation in this study is voluntary, and concerns only the collection and use of your medical data collected during your hospitalization. If you refuse, this will have no impact on your post-COVID-19 care and follow-up.

[**1.** **Objectives of the OXYCOVID study** 2](#_Toc190026112)

[**2.** **Process of the study** 2](#_Toc190026113)

[**3.** **Expected benefits** 3](#_Toc190026114)

[**4.** **Your personnal data** 3](#_Toc190026115)

[**5.** **Your participation** 4](#_Toc190026116)

[**6.** **Costs** 4](#_Toc190026117)

[**7.** **Contacts** 4](#_Toc190026118)

# **Objectives of the OXYCOVID study**

We are writing to you because you have recently been hospitalized at the *Infirmerie Protestante* of Lyon following COVID-19 pneumopathy.

COVID-19 is a complex disease in which the interaction of the virus with target cells, the action of the immune system and the body's systemic response are closely linked.

Various studies have observed that COVID-19 causes the death of infected cells, activation of the innate immune response and secretion of pro-inflammatory substances known as cytokines. All these processes increase oxidative stress, a major contributor to the initiation and development of viral infections. In the case of COVID-19, it has been observed that the interaction between increased cytokine levels in the blood and oxidative stress could play an important role in the severity of symptoms and patient outcome.

Since the start of the pandemic, we have observed that clinical symptoms are highly variable from one patient to the next, and that the course of the disease remains unpredictable. At present, we have no specific blood biomarkers to determine the severity of the infection and predict the risk of worsening in patients' clinical condition.

The main aim of this study is therefore to assess the link between the clinical course of patients hospitalized for COVID-19 infection and their oxidative stress levels.

# **Process of the study**

The OXYCOVID study is a retrospective study based solely on medical information collected during your last hospitalization at the *Infirmerie Protestante*.

In practice, we are simply asking you to collect data from your medical records, so that we can combine them with those of other patients who have had similar treatment to yours. These data will then be statistically processed to answer the questions of this study. As this research is based solely on the data available in your medical file, your participation in this study will not require any additional medical visits or examinations, and will in no way modify your current care and treatment.

# **Expected benefits**

If you agree to participate in this study, you will not derive any direct benefit.

However, the information gathered in this study will be used to improve management and predict the risk of worsening in future COVID-19 patients.

# **Your personnal data**

In accordance with the new regulations for the protection of personal data, we would like to inform you about the use of your personal data.

The medical data collected during your hospitalization will be processed electronically, and entered into an electronic database (eCRF). You will be identified in the database by a patient number associated with the first two letters of your surname and first name.

The Clinical Research Unit of the *Infirmerie Protestante* de Lyon undertakes to comply with the regulatory requirements of the French Data Protection Act and the *Commission Nationale de l'Informatique et des Libertés* (CNIL) and European Regulation 2016/679 on the protection of individuals with regard to the processing of personal data and on the free movement of such data. The processing of your personal data will also be carried out in strict compliance with the applicable reference methodology published by the CNIL.

In accordance with the provisions of the French Data Protection Act (article 40, *loi Informatique et Liberté* modified on January 6^th^ 1978), and the regulations governing the protection of personal data, you have a right of access, rectification and opposition which you may exercise at any time. The data collected about you before you withdrew your consent cannot be deleted and will continue to be processed under the conditions laid down by the study. Your right to withdraw consent may be exercised directly with the coordinating investigator, Doctor RASPADO Olivier, or through the Clinical Research Unit.

Your medical information collected as part of this study will be kept until the final report or publication of the research results, and will then be archived in accordance with regulations, i.e. for a minimum of 15 years after the end of the research or its early termination.

**Key messages :**

- All documents in which your identity appears will be kept by the doctor treating you and will remain strictly confidential.

- You will be identified by a patient number that will allow your identity to be masked and your data to be entered into a database.

- You have a right of access, rectification, opposition and limitation of data processing.

# **Your participation**

Your participation in this clinical study is voluntary. You will be given a period of time to respond before informing us of your interest in participating.

If you agree to participate, you may withdraw your consent at any time and freely stop your participation in the study without giving any reason. This interruption will have no impact on your post-COVID-19 care and follow-up. Your data will be stored and analyzed until you withdraw from the study, unless you object.

On the other hand, if you do not wish to participate in the study, this will have no impact on your post-COVID-19 management and follow-up.

# **Costs**

You will not be compensated for your participation in the OXYCOVID study.

Your participation is entirely voluntary.

# **Contacts**

If you have any questions about the study or any problems, please contact:

Doctor **RASPADO Olivier**, investigating physician for this study: +33 (**0)4.72.00.71.65.**

The **clinical research unit** : +33 (**0)4.26.29.79.27.**
