# Supplementary material for: Oxidative Stress Markers and Prediction of Severity With a Machine Learning Approach in Hospitalized Patients With COVID-19 and Severe Lung Disease: Observational, Retrospective, Single-Center Feasibility Study
Source: JMIR Form Res. 2025 Apr 11;9:e66509. doi: 10.2196/66509 (PMC12007842; doi:10.2196/66509)
Supplement: Multimedia Appendix 2 [file formative-v9-e66509-s002.docx]

Appendix

The following table detailed the SVM results related to the 28 patients included in the study.

Examples of interpretation:

- Clinically, patient no. 15 was initially classified as grade 0, he was asymptomatic. Its biological analysis profile was completely satisfactory (high thiols = 7.2, high zinc, Cu/zn ratio = 1). The SVM model effectively classifies it as grade 0 with a probability of 99.1%.
- Clinically, patient no. 11 was initially classified as grade 2, he was strongly symptomatic. Its biological analysis profile was very disturbed (average thiols = 4.6, very low zinc, very high Cu/zn ratio = 2.5). The SVM model classifies it in grade 2 with a probability of 68.1%.

Table: SVM results related to the 28 patients ; OS biomarkers observed and probabilities of belonging to the grades 0, 1 or 2.

| Patient | Zinc | cuivre | Cu/Zn | sélénium | ac. urique | CRPus | LDLox | G.peroxydase | G.réductase | Thiols | Clinical Grade | Prob[0] | Prob[1] | Prob[2] | Most Likely Grade |
| --- | --- | --- | --- | --- | --- | --- | --- | --- | --- | --- | --- | --- | --- | --- | --- |
| 1 | 589 | 1384 | 2,34 | 83 | 313 | 29 | 90 | 770 | 10 | 4,3 | 2 | 7,9% | 25,6% | 66,6% | 2 |
| 2 | 675 | 1436 | 2,12 | 51 | 98 | 315 | 100 | 349 | 12,4 | 4 | 2 | 6,3% | 25,9% | 67,8% | 2 |
| 3 | 320 | 988 | 3,08 | 40 | 533 | 127 | 27 | 487 | 9,9 | 4,6 | 2 | 6,5% | 25,9% | 67,6% | 2 |
| 4 | 459 | 1642 | 3,57 | 43 | 292 | 172,9 | 69 | 416 | 11,9 | 4,5 | 2 | 2,6% | 65,8% | 31,6% | 1 |
| 5 | 572 | 1293 | 2,26 | 72 | 243 | 7,95 | 70 | 341 | 6,5 | 4,8 | 1 | 31,2% | 64,1% | 4,6% | 1 |
| 6 | 442 | 1351 | 3,05 | 43 | 225 | 90,6 | 72 | 247 | 12,2 | 4,4 | 1 | 4,6% | 79,1% | 16,3% | 1 |
| 7 | 595 | 1313 | 2,2 | 75 | 266 | 206,3 | 91 | 602 | 9,7 | 4,1 | 1 | 5,9% | 23,4% | 70,7% | 2 |
| 8 | 649 | 1185 | 1,82 | 50 | 366 | 1,61 | 26 | 234 | 8,6 | 5,7 | 0 | 88,8% | 9,6% | 1,5% | 0 |
| 9 | 534 | 1119 | 2,09 | 47 | 367 | 388,85 | 59 | 200 | 10,6 | 2,4 | 1 | 0,4% | 91,6% | 8,0% | 1 |
| 10 | 362 | 1708 | 4,71 | 48 | 376 | 388,14 | 78 | 437 | 12,8 | 2,9 | 1 | 0,1% | 82,2% | 17,7% | 1 |
| 11 | 352 | 883 | 2,5 | 37 | 159 | 409,25 | 28 | 357 | 9,7 | 4,6 | 2 | 6,0% | 25,9% | 68,1% | 2 |
| 12 | 918 | 1424 | 1,55 | 56 | 313 | 29,71 | 69 | 457 | 9,4 | 4,5 | 0 | 75,6% | 13,8% | 10,6% | 0 |
| 13 | 527 | 1189 | 2,25 | 69 | 272 | 3,18 | 47 | 502 | 9,1 | 5,7 | 0 | 47,7% | 45,9% | 6,3% | 0 |
| 14 | 854 | 858 | 1 | 72 | 352 | 1,05 | 32 | 390 | 9,4 | 7,2 | 0 | 99,1% | 0,6% | 0,3% | 0 |
| 15 | 488 | 981 | 2,01 | 58 | 431 | 77,9 | 32 | 442 | 7,7 | 4,7 | 1 | 23,3% | 63,3% | 13,5% | 1 |
| 16 | 596 | 1095 | 1,84 | 71 | 437 | 16,69 | 61 | 375 | 11,3 | 4 | 1 | 26,7% | 66,5% | 6,8% | 1 |
| 17 | 754 | 1599 | 2,12 | 58 | 251 | 34 | 56 | 256 | 8,7 | 4 | 0 | 53,5% | 41,2% | 5,3% | 0 |
| 18 | 524 | 1311 | 2,5 | 52 | 261 | 105,1 | 38 | 424 | 8,5 | 4,7 | 1 | 15,2% | 76,9% | 7,9% | 1 |
| 19 | 719 | 1351 | 1,88 | 36 | 499 | 390,18 | 22 | 181 | 11,2 | 3,7 | 1 | 7,3% | 90,9% | 1,9% | 1 |
| 20 | 719 | 1134 | 1,57 | 77 | 170 | 10,04 | 44 | 389 | 8,8 | 5,7 | 0 | 86,0% | 12,3% | 1,7% | 0 |
| 21 | 629 | 1384 | 2,2 | 55 | 306 | 158,29 | 55 | 499 | 11,8 | 5,6 | 1 | 26,6% | 58,5% | 14,9% | 1 |
| 22 | 731 | 1291 | 1,76 | 78 | 262 | 9,58 | 127 | 516 | 9,7 | 5,9 | 0 | 60,9% | 16,3% | 22,9% | 0 |
| 23 | 564 | 1258 | 2,23 | 50 | 211 | 158,57 | 67 | 419 | 9,1 | 3,2 | 2 | 5,7% | 37,4% | 56,9% | 2 |
| 24 | 609 | 995 | 1,63 | 35 | 692 | 125,55 | 66 | 227 | 8,6 | 5,5 | 0 | 62,2% | 16,6% | 21,2% | 0 |
| 25 | 635 | 1617 | 2,54 | 53 | 160 | 25,8 | 60 | 492 | 8,7 | 4,6 | 1 | 26,7% | 58,4% | 14,9% | 1 |
| 26 | 430 | 1190 | 2,76 | 46 | 186 | 153,25 | 33 | 499 | 10,1 | 3,4 | 1 | 1,6% | 81,1% | 17,4% | 1 |
| 27 | 557 | 1752 | 3,14 | 59 | 453 | 109 | 56 | 397 | 7,8 | 5,2 | 1 | 16,5% | 79,2% | 4,3% | 1 |
| 28 | 568 | 1692 | 2,97 | 53 | 741 | 217 | 70 | 394 | 10,1 | 4,4 | 1 | 4,2% | 78,9% | 16,9% | 1 |
